# A new sawfly of Megalodontesidae (Insecta, Hymenoptera, Pamphilioidea) with pectinate antennae from the Early Cretaceous of China

**DOI:** 10.3897/zookeys.893.38512

**Published:** 2019-12-02

**Authors:** Yimo Wang, Mei Wang, Chungkun Shih, Alexandr P. Rasnitsyn, Jun Yao, Dong Ren, Taiping Gao

**Affiliations:** 1 College of Life Sciences and Academy for Multidisciplinary Studies, Capital Normal University, 105 Xisanhuanbeilu, Haidian District, Beijing 100048, China Capital Normal University Beijing China; 2 Key Laboratory of Forest Protection, State Forestry Administration, Research Institute of Forest Ecology, Environment and Protection, Chinese Academy of Forestry, Beijing 100091, China Research Institute of Forest Ecology, Environment and Protection, Chinese Academy of Forestry Beijing China; 3 Department of Paleobiology, National Museum of Natural History, Smithsonian Institution, Washington, DC, 20013-7012, USA Smithsonian Institution Washington United States of America; 4 Palaeontological Institute, Russian Academy of Sciences, 117868, Moscow, Russia Palaeontological Institute, Russian Academy of Sciences Moscow Russia; 5 The Natural History Museum, Cromwell Road, London SW7 5BD, UK The Natural History Museum London United Kingdom; 6 Institute of Apicultural Research of CAAS, No. 1 Beigou Xiangshan, Haidian District, Beijing 100093, China Institute of Apicultural Research of CAAS Beijing China

**Keywords:** ramified antennae, Symphyta, taxonomy, Yixian Formation

## Abstract

A new sawfly of Megalodontesidae, *Jibaissodes peichenae***sp. nov.**, is described from the Lower Cretaceous Yixian Formation of Northeastern China. It is established mainly based on the pectinate antenna comprising 42 flagellomeres and the proximal 28 bearing apical rami, which gradually shorten in length toward the apex of the flagellum. The pterostigma of the forewing is infuscated apically and on the hind wing, vein 1-Rs is nearly equal to 1r-m and slightly shorter than 1-M. The first tergum is widely excised posteriorly and roundly protruding laterally alike in *Megalodontes*. This find supports that pectinate antennae in extant sawflies of Megalodontesidae originated at least during or before the Early Cretaceous.

## Introduction

Megalodontesidae is a small extant family with only one extant genus comprising 42 species and 12 fossil genera with 23 species (Taeger et al. 2018). Megalodontesidae comprises four subfamilies: three extinct subfamilies, Archoxyelydinae Wang, Rasnitsyn & Ren, 2013, Decorisiricinae Wang, Rasnitsyn, Shih, Sharkey & Ren, 2015, and Praesiricinae Rasnitsyn, 1968 and one extant subfamily, Megalodontesinae Konow, 1897. The sole extant genus, *Megalodontes* Latreille, 1803, which is distributed in temperate regions of the Palaearctic (Benson 1968; Goulet 1993; Blank et al. 2001; Taeger et al. 2010), and an extinct genus, *Jibaissodes* Ren, Lu, Guo & Ji, 1995, are characterized by having saw-like or comb-like ramified antennae.

Among extant symphytan insects, ramified antennae are present in various taxa, i.e., in Diprionidae, Pergidae, Tenthredinidae, and Megalodontesidae (Gao et al. 2016). Fossil records of ‘Symphyta’ with ramified antennae are very rare. Up to date, only two species with ramified antennae have been reported: *Jibaissodes bellus* Gao, Shih Labandeira, Santiago-Blay, Yao & Ren, 2016 with simply ramified antennae from the Lower Cretaceous Yixian Formation, and *Atefia rasnitsyni* Krogmann, Engel, Bechly & Nel, 2012 with the biflabellate antennae from the Lower Cretaceous Crato Formation, assigned to the superfamily Tenthredinoidea s. str. Latreille, 1802. The occurrence of biflabellate antennae in *A. rasnitsyni* has been suggested to indicate the antiquity of insect usage of long-range female attractants (Krogmann et al. 2013).

In this paper, we describe *Jibaissodes peichenae* sp. nov. based on a well-preserved specimen from the mid-Lower Cretaceous Yixian Formation of northeastern China. The new species with distinctly pectinate antennae contributes additional important morphological characters of the family Megalodontesidae.

## Material and methods

For the paper we examined the holotypes of *Jibaissodes peichenae* sp. nov. (specimen no. CNU-HYM-LB2018033, part and counterpart) and *Jibaissodes bellus* Gao, Shih, Labandeira, Santiago-Blay, Yao & Ren, 2016 (specimen no. CNU-HYM-LB2011009, part and counterpart), which are housed in the Key Laboratory of Insect Evolution and Environmental Changes at the Capital Normal University, Beijing, China (**CNUB**; Dong Ren, curator). Both were collected from the mid-Lower Cretaceous Yixian Formation of Liaoning Province of China. The holotype of *Jibaissodes giganteus* Ren, Lu, Guo & Ji, 1995 (specimen no. BL92105, part, housed in the Geological Museum of China, Beijing, China, Jun Yao, curator) was also examined and redrawn for this paper. It was collected from the Lower Cretaceous Lushangfen Formation of Beijing of China.

The specimens were examined and photographed, either dry or wetted with 95% ethanol, by using a Nikon SMZ25 with an attached camera system. Line drawings were prepared using Adobe Illustrator CC and Adobe Photoshop CC graphics software. The wing venation nomenclature used in the paper was modified after Rasnitsyn (1969, 1980).

## Taxonomy

### Genus Jibaissodes

Taxon classificationAnimaliaHymenopteraPamphilioidea

Ren, Lu, Guo & Ji, 1995

78853A0E-0EF8-5119-8908-87A4A3E15C01

#### Emended diagnosis.

Mesonotum large, notauli strongly impressed, tapering to acutely rounded base. Forewing veins 1-Rs and 1-M nearly in straight line; 1r-rs present, shorter than 2r-rs; 1r-rs reclival and 2r-rs proclival; 1m-cu near base of cell 2rm; 1cu-a at base of cell 1mcu, connecting to juncture of M+Cu; cell 1mcu small, nearly rectangular; cell 2rm longer than cell 3rm. Hind wing with 1-Cu nearly perpendicular to cu-a; 1A and 2A strongly curved.

#### Type species.

*Jibaissodes giganteus* Ren, Lu, Guo & Ji, 1995

#### Emended description.

**Figure 1.**
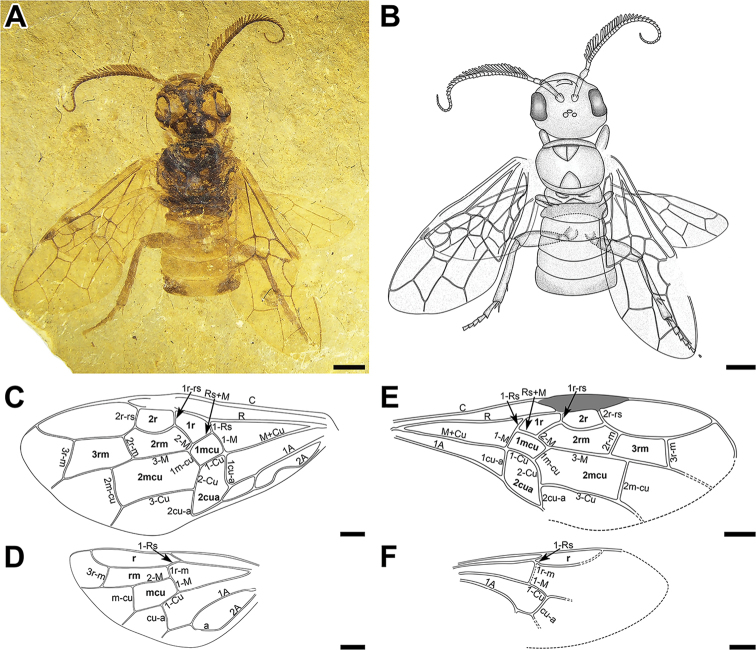
**A–D***Jibaissodes peichenae* sp. nov., holotype, part: **A** photograph of complete specimen **B** line drawing of complete specimen **C** line drawing of forewing **D** line drawing of hind wing **E, F***Jibaissodes giganteus*, holotype: **E** line drawing of forewing **F** line drawing of hind wing. Scale bars: 2 mm (**A, B**), 1 mm (**C–F**).

Fossil incomplete: head appendages, pronotum, legs, abdomen and supposedly posterior parts of wings missing. Head moderately large, near oval; compound eyes large; ocelli not visible. Mesonotum broader than long; mesoscutum small, with distinct medial line and impressed notauli; mesoscutellum indistinct; metanotum narrow and long; metascutum with cenchri present but indistinct. Forewing (Fig. 1E) with Sc absent; pterostigma long, completely infuscated; 1-Rs reclival, slightly shorter than 1-M; 1-M long and straight; Rs-M juncture nearly straight; Rs+M straight, forming a nearly straight line with 2-M, nearly perpendicular to 1-M; 1r-rs slightly shorter than 2r-rs; 2r-rs distinctly proclival, positioned near distal 0.75 of pterostigma; 1cu-a straight, distinctly bent towards wing tip; M+Cu straight; 1-Cu and 2-Cu curved, 1-Cu nearly equal to 2-Cu; 2r-m straight and reclival; 3r-m straight towards wing tip; 1A slightly curved. Costal cell slightly widened before point of origin of Rs; cell 2rm distinctly longer than cell 3rm, cell 3rm trapezoid; cell 1mcu small, nearly rectangular; cell 2mcu large, pentagonal. Hind wing (Fig. 1F) with 1-Rs very short, 1r-m straight; 1r-m and 1-M forming a straight line. M+Cu slightly bent; 1-Cu slightly curved, nearly perpendicular to cu-a; cu-a nearly straight; vein 1A strongly arched.

#### Other species included.

*Jibaissodes bellus* Gao, Shih, Labandeira, Santiago-Blay, Yao & Ren, 2016; *Jibaissodes peichenae* sp. nov.

### Jibaissodes peichenae

Taxon classificationAnimaliaHymenopteraPamphilioidea

Wang, Shih & Gao sp. nov.

B1D59903-AC26-539C-9154-8FF548470CBE

http://zoobank.org/D752BA0D-21EF-44AB-BBC7-B9E4279A6883

Figs 1 , 2 , 3

#### Type material.

Holotype, specimen no. CNU-HYM-LB2018033p/c, part and counterpart.

#### Diagnosis.

Antenna pectinate, with 42 flagellomeres, flagellum longer than head width, proximal 28 flagellomeres with apical rami, rami gradually shortening in length toward apex of flagellum; scape almost 3 times as long as first flagellomere. Anterior margin of pronotum round, with weakly concave posterodorsal margin. Forewing with pterostigma infuscated apically; vein 1cu-a strongly curved. Hind wing vein 1-Rs nearly equal to 1r-m, slightly shorter than 1-M.

#### Description.

***Body*** (Figs 1A, B, 3A). Fossil incomplete: distal abdominal segments missing, part of the legs invisible. Middle and surrounding regions of compound eyes and part of posterior head pale, remainder of head dark. Thorax and legs entirely or predominantly dark. First tergum except hind margin and fifth tergum laterally dark. Body about 14.9 mm (but distal abdominal segments missing), antenna 11.4 mm long; forewing about 13.6 mm in length, maximum width 6.0 mm; hind wing about 8.5 mm in length, maximum width 3.8 mm.

**Figure 2.**
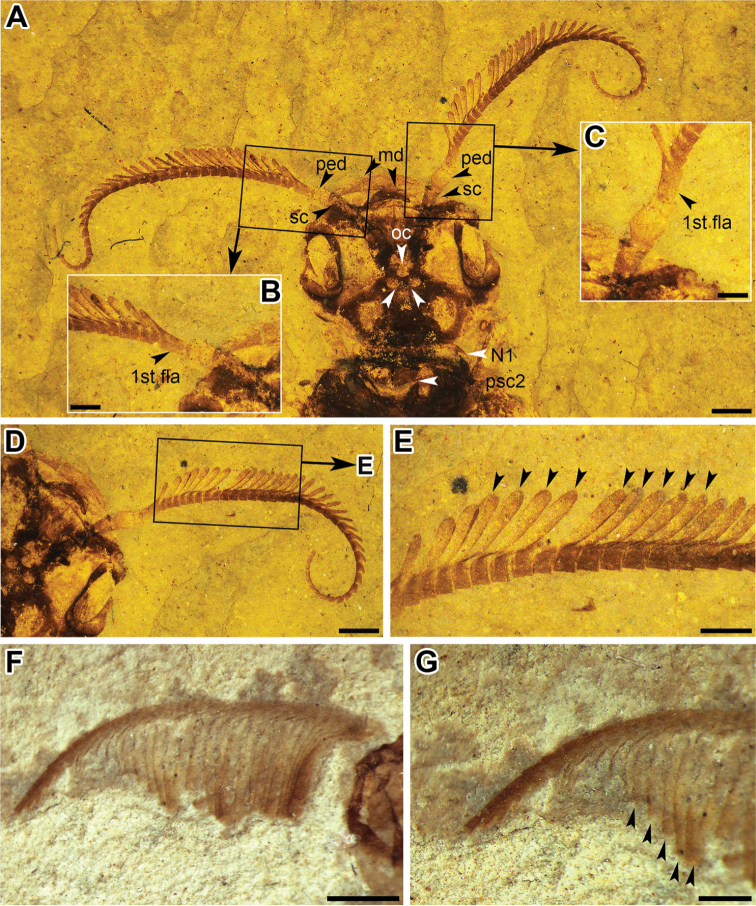
**A–E***Jibaissodes peichenae* sp. nov., holotype, part: **A** head **B** basis of left antenna **C** basis of right antenna **D** right antenna **E** basal to middle section of right antenna **F, G***Jibaissodes bellus*, holotype: **F** right antenna **G** apical portion of right antenna. Abbreviations: md = mandible, sc = scape, ped = pedicel, oc = ocelli, 1st fla = 1st flagellomere, N1 = pronotum, psc2 = mesoscutum. Scale bars: 1 mm (**A, D, F, G**); 0.5 mm (**B, C, E**).

***Head*.** Large, about as wide as thorax. Head (Fig. 2A) 5.3 mm wide and 4.6 mm long, almost circular. Compound eyes 1.8 mm long, 1.1 mm wide; right mandible large, bent and sickle-shaped; antenna (Fig. 2D, E) with 42 flagellomeres, basal 28 flagellomeres with rami extending from apicolateral angle, apical 14 flagellomeres without distinct rami, longest ramus about 0.8 mm in length, longest ramus slightly longer than total length of following three flagellomeres; rami gradually shortening in length toward apex of flagellum, first flagellomere much shorter than scape, 0.3 mm wide, 0.5 mm long; scape 1.4 mm long, maximum 0.4 mm in width; pedicel 0.8 mm long, maximum 0.5 mm wide.

**Figure 3.**
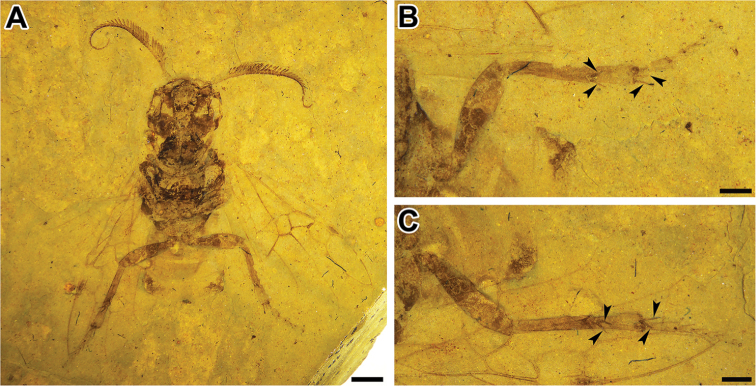
*Jibaissodes peichenae* sp. nov., holotype, counterpart **A** complete specimen **B** right hind leg **C** left hind leg. Arrows indicate spurs. Scale bars: 2 mm (**A**); 1 mm (**B, C**).

***Thorax*.** Maximum width 4.9 mm; pronotum short, apex round, with weakly concave posterodorsal margin. Mesoscutum large, with medial line and notauli strongly impressed, tapering to acutely rounded base; mesoscutum without line to mesoscutellum; mesoscutellum tapering to acutely sharp apex; metascutum with cenchri present and small. Metatibia (Fig. 3B, C) with two preapical (near distal 0.7 of length) and two apical spurs; metabasitarsus long but shorter than remaining tarsomeres combined; metafemur, metatibia, and metabasitarsus lengths 3.4 mm, 4.6 mm, and 1.1 mm, respectively.

***Abdomen*.** Five visible segments preserved; maximum width about 5.3 mm; first tergum medially undivided, posteriorly widely excised, laterally roundly protruding, medio-anteriorly dark; fifth tergum laterally dark.

***Forewing*.** (Fig. 1B, C) Wing membrane mostly hyaline with weakly infuscated bands covering base and middle regions of forewing, surrounding veins R, M+Cu, 1r-rs, 2-M, m-cu, and 2-Cu; R slightly thickened before pterostigma; pterostigma infuscated apically; 1-Rs (0.5 mm long) short and reclival; 1-M (1.0 mm long) long and slightly curved, about twice as long as 1-Rs; Rs+M straight; 1r-rs length 0.7 mm, slightly shorter than 2r-rs, 0.8 mm long; 2r-rs slightly proclival, positioned near 3/4 pterostigma; 1cu-a distinctly bent towards wing base, 1.4 mm in length; M+Cu nearly straight; 1m-cu 0.8 mm long, 0.7 times as long as 1-Cu (1.1 mm long); 1-Cu nearly equal to 2-Cu (1 mm long) in length; 2r-m slightly curved and reclival; 3r-m straight towards wing tip; 2A with shallow arch midway before 1a-2a cross-vein. Costal cell widened at point of origin of 1-Rs; cell 1r nearly equal in size to cell 2r; cell 2rm longer than cell 3rm, cell 3rm trapezoid; cell 1mcu small, nearly rectangular; cell 2mcu large, pentagonal.

***Hind wing*.** (Fig. 1B, D) 1-Rs (0.5 mm long) nearly equal to 1r-m (0.5 mm long), shorter than 1-M (0.7 mm long), 1r-m straight; 1r-m and 1-M forming a nearly straight line; 3r-m present, 0.9 mm in length, strongly bent towards wing tip; 1m-cu (1.0 mm long) and cu-a (1.1 mm long) present, longer than 3r-m, cu-a distinctly bent; M+Cu nearly straight; vein 1A strongly arched.

***Gender.*** Unknown.

#### Remarks.

The species can be assigned to *Jibaissodes* according to the following diagnostic characters: forewing vein 1r-rs present, shorter than 2r-rs, cell 2rm longer than 3rm and cell 1mcu small. *Jibaissodes* was described from a poorly preserved fossil specimen with a left and a right forewing, a right hind wing, and parts of head and thorax (Ren et al. 1995). *Jibaissodes peichenae* sp. nov. is distinctly differentiated from the type species of *J. giganteus* by the following characters: forewing with pterostigma not completely infuscated; forewing vein 3r-m distinctly longer than 2r-m (about 1.7 times as long as 2r-m); 1cu-a distinctly curved; and hind wing vein 1-Rs nearly equal to 1r-m. Furthermore, *J. peichenae* is distinguished from *J. bellus* by the following characters: antenna pectinate, rami short (vs rami long in *J. bellus*); forewing with pterostigma infuscated apically (vs infuscated medially and apically in *J. bellus*).

#### Distribution.

Huangbanjigou, near Chaomidian Village, in Shangyuan County, adjacent to Beipiao City, in Liaoning Province of China. Collected from the mid-Lower Cretaceous Yixian Formation, dated as latest Barremian to earliest Aptian, 125 Ma (Ren et al. 1995; Wang et al. 2015).

#### Etymology.

The species epithet is dedicated to Miss Peichen Yao, the daughter of Dr Jun Yao, the specimen donator.

## Discussion

*Jibaissodes peichenae* sp. nov. is assigned to Megalodontesidae by two features typical for Megalodontesidae: undivided first tergum and absent Sc (Benson 1968; Goulet 1993; Wang et al. 2016). In addition, *J. peichenae* can be attributed to Megalodontesinae, by the pectinate antennae as a derived character state. Other fossil Megalodontesidae, i.e., Decorisiricinae, Archexyelinae, and Praesiricinae, have a synantennomere 3 (Blank et al. 2013) like the ancestral Xyelidae and a number of fossil taxa, which represents an ancestral character of Hymenoptera (e.g., Rasnitsyn 1996). The semicircular distal excision and the lateral round protuberances of the first abdominal tergum is a putative apomorphy of *Jibaissodes* + *Megalodontes*, since protrusions are obviously absent in Decorisiricinae, Archexyelinae, and Praesiricinae. On the forewing, M+Cu is straight, while it is curved in Archexyelinae and Praesiricinae.

All fossil megalodontesids share as a plesiomorphy of a curved vein 2A on the forewing (Wang et al. 2015, 2016). The almost straight 2A of the extant *Megalodontes* is an apomorphy of this taxon (Taeger 2002; Taeger et al. 2014). Therefore, we treat *Jibaissodes* and *Megalodontes* as separate groups. Although *J. peichenae* sp. nov. shares two features with *Megalodontes* (pectinate antennae and laterally protruding, medio-anteriorly dark first tergum; Taeger 2002), it is impossible to evaluate the coloration of head, pronotum, and mesonotum of *J. peichenae* as preserved on fossil with certainty. We need more fossil specimens to address whether *Jibaissodes* has close affinities with *Megalodontes*.

Since weak sexual dimorphism has been observed within several groups of *Megalodontes* having antennae with relatively long rami in males and slightly short rami in females (Taeger 2002), and thereby, we cannot rule out the possibility that these two species, *J. peichenae* with long ramified antennae and *J. bellus* with short ramified antennae, might be different sexes of the same species. However, *J. peichenae* differs from *J. bellus* also in the following characters: pterostigma of forewing infuscated apically and, particularly, the excised posterior edge of first tergum, which is more similar to *Megalodontes* (Taeger 2002; Taeger et al. 2014). Given that the holotype of *J. peichenae* lacks the tip of the abdomen, it is impossible to properly ascertain whether the two should be considered as the same species. Therefore, we treat them as separate for now, pending future finds of additional fossil specimens to confirm the relationship between these two species.

## Supplementary Material

XML Treatment for Jibaissodes

XML Treatment for Jibaissodes peichenae
